# The Human Carbonic Anhydrase II in Platelets: An Underestimated Field of Its Activity

**DOI:** 10.1155/2018/4548353

**Published:** 2018-06-28

**Authors:** Maciej Jakubowski, Ewa Szahidewicz-Krupska, Adrian Doroszko

**Affiliations:** Department of Internal Medicine, Occupational Diseases and Hypertension, Wroclaw Medical University, Borowska 213, 50-556 Wroclaw, Poland

## Abstract

Carbonic anhydrases constitute a group of enzymes that catalyse reversible hydration of carbon dioxide leading to the formation of bicarbonate and proton. The platelet carbonic anhydrase II (CAII) was described for the first time in the '80s of the last century. Nevertheless, its direct role in platelet physiology and pathology still remains poorly understood. The modulation of platelet CAII action as a therapeutic approach holds promise as a novel strategy to reduce the impact of cardiovascular diseases. This short review paper summarises the current knowledge regarding the role of human CAII in regulating platelet function. The potential future directions considering this enzyme as a potential drug target and important pathophysiological chain in platelet-related disorders are described.

## 1. Carbonic Anhydrases

Carbonic anhydrases constitute a group of zinc containing lyases, classified, according to the Enzyme Catalogue to EC 4.2.1.1., into the lyases subclass “carbon-oxygen lyases” and subclasses “hydrolyses” [1]. They catalyse reversible hydration of carbon dioxide to form bicarbonate ion and proton. Carbonic anhydrase is present in prokaryotic and eukaryotic cells. Genetically they belong to seven subgroups (*α*, *β*, *γ*, *δ*, *ζ*, *η*, *θ*) that are not evolutionarily related [2–4]. There are 15 carbonic anhydrase isoforms in humans (belonging to the *α* subgroup) [5]. They occur in various tissues in the cytoplasm, cell membrane, and mitochondria, or as extracellular enzymes (e.g., breast milk) [6]. Carbonic anhydrases (CAs) are almost ubiquitously present in human cells. The members of the CAs family play a role in pH regulation, gas exchange, and ion transport, as well as urine acidification, cerebrospinal fluid secretion, ocular fluid production, bone resorption, fatty acid metabolism, testicular fluid production, and many others [7].

Numerous diseases result from inappropriate function of carbonic anhydrases. There are scarcely several case reports regarding CAs deficiency; nonetheless they constitute a valuable source of knowledge. The erythrocyte CAI deficiency has no clinically relevant consequences [8], but CAII deficiency usually results in osteoporosis, renal tubular acidosis, and brain calcification [9, 10]. CAIII deficiency in skeletal muscles seems to play an import role in pathogenesis of myasthenia gravis [11, 12]. The carbonic anhydrase VA deficiency may present as early-onset liver failure with hyperammonemia, hyperlactatemia, and ketonuria [13]. CAXII deficiency may promote hyponatremic dehydration and rhabdomyolysis after intense physical exercises [14]. The effectiveness of carbonic anhydrase may be also altered by autoantibodies. Ignaki et al. demonstrated in 1991 that anti-carbonic anhydrase autoantibodies (aCAAs) are present in serum of 32% patients with systemic lupus erythematosus, in 21% of patients with Sjögren's syndrome, but not in healthy volunteers. They showed that aCAAs have affinity to epidermal cells, hair follicles, sweat glands, and renal tubular cells [15]. Moreover, in 2007, it was presented that also in rheumatoid arthritis there are present aCAAs in serum, showing affinity to bind to carbonic anhydrase III in synovial membranes [16]. Up to date aCAAs have been described in patients with rheumatoid disorders (rheumatoid arthritis, Behçet's disease, lupus erythematosus, polymyositis, systemic sclerosis, and Sjögren syndrome) [16–18], digestive tract disorders (idiopathic chronic pancreatitis, primary biliary cirrhosis, autoimmune cholangitis, and gastric cancer) [18, 19], endometriosis [18], Grave's disease [20], acute myeloid leukaemia [21], renal tubular acidosis [22] (significant influence on pathogenesis proved in the animal model of Sjögren's syndrome [23]), and end-stage kidney disease [24]. Moreover, autoantibodies against carbonic anhydrase II were proved to play an important role in pathogenesis of retinopathy [25] and these against CAVI seem to induce dry eye syndrome in Sjögren's syndrome [26, 27]. Interestingly, the autoantibodies against carbonic anhydrase II might be produced in humans due to cross-reactivity with carbonic anhydrase of Helicobacter pylori [28].

Carbonic anhydrases (CAs) have become an interesting enzyme for clinicians when first drugs inhibiting this enzyme established promising group of diuretics. Acetazolamide was introduced into clinical practice in 1956 as a first nonmercurial diuretic. Among “classic” carbonic anhydrase inhibitors also methazolamide, ethoxzolamide, and dichlorphenamide were applied in congestive heart failure treatment. Nowadays, more preferable are the next-generation diuretics (loop, thiazide, thiazide-like diuretic, and aldosterone antagonists), but most of them still exhibit different magnitude of carbonic anhydrase inhibition [38]. Acetazolamide remains a useful drug in case of intracranial hypertension [39], improving sleep quality (reducing the apnea-hypopnea index in both obstructive sleep apnea and healthy trekkers) [40] and reducing the risk of mountain sickness at high altitudes [41]. Topical dorzolamide and brinzolamide and in some cases systemic acetazolamide are commonly used for reduction of intraocular pressure in glaucoma [42]. Topiramate and zonisamide are anticonvulsants with a multiple mechanism of action among which is also carbonic anhydrase inhibition. Not only does this effect seem to be important in the main (antiepileptic) activity of this drug, but also it might result in weight loss. It is regarded as a side effect but may be the basis for designing the new antiobesity drugs [38]. Moreover, acetazolamide is also proved to be effective in seizure treatment [43]. Induced by hypoxia, overexpression of carbonic anhydrase IX in neoplastic cells may produce acidosis and thus reduce the effectiveness of chemotherapies [38]. Inhibition of carbonic anhydrase IX improves effects of cisplatin in small cell lung cancer [44].

## 2. Carbonic Anhydrase II

**Figure 1:**
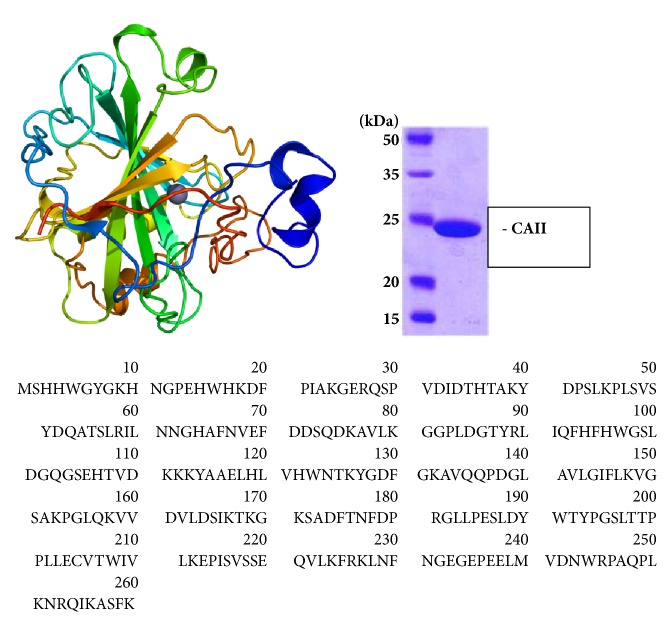
The structure and amino acid sequence of the human carbonic anhydrase II [29].

Carbonic anhydrase II (CAII) is a cytoplasmic enzyme with a very high affinity for sulphonamides and high catalytic activity (for structure see Figure 1.). It is one of the fastest, working enzymes in the human body, 10^6^ cycles of enzyme per second [45]. CAII is present in numerous tissues (e.g., erythrocytes, eye, gastrointestinal tract, bone osteoclasts, kidney, lung, testis, and brain) and it constitutes a possible drug target in some diseases (glaucoma, oedema, epilepsy, and attitude sickness). However, so far it has been mostly studied in the red blood cells and has been extracted from them for the purpose of* in vitro* studies [5].

The gene for human CAII is located on the chromosome 8q22 [46]. The diseases associated with CAII include the following: Autosomal Recessive Osteopetrosis Type 3 with Renal Tubular Acidosis (ARO3, OPTB3), which commonly manifests in early infancy with macrocephaly, feeding difficulties, evolving blindness and deafness, bone marrow failure, severe anaemia, and hepatosplenomegaly. Deafness and blindness are generally thought to represent effects of pressure on nerves. OPTB3 is associated with renal tubular acidosis and cerebral calcification (marble brain disease) and in some cases with mental retardation [47]. Among its related pathways is vitamin D-receptor mediated regulation of genes involved in osteoporosis [48].

Numerous compounds have been demonstrated to modify carbonic anhydrase activity. They are physiological substances, drugs currently used in clinical practice and other chemicals tested* ex vivo* in laboratories. Potential CAII activators physiologically present in human organism include biogenic amines (histamine, catecholamines, and serotonin) and amino acids (phenylalanine and histidine) [49]. Most drugs inhibiting carbonic anhydrase are sulphonamides (over 20 FDA approved drugs including diuretics like hydrochlorothiazide, indapamide, chlortalidone, and furosemide) [50, 51]. There is a wide variety of substances testes in laboratory conditions for their CA activatory and inhibitory properties [5, 52].

The aim of this review article was to collect available data regarding studies on carbonic anhydrase in platelets and concerning the influence of carbonic anhydrase regulators on platelet function. Taking into account limited number of studies directly analyzing carbonic anhydrase properties in platelets, a secondary objective was to analyze the involvement of drugs regulating CAII in platelet pathophysiology and to discuss hypothetical contribution of platelet carbonic anhydrase in these drug-platelet interactions.

## 3. Platelet Carbonic Anhydrase

**Figure 2:**
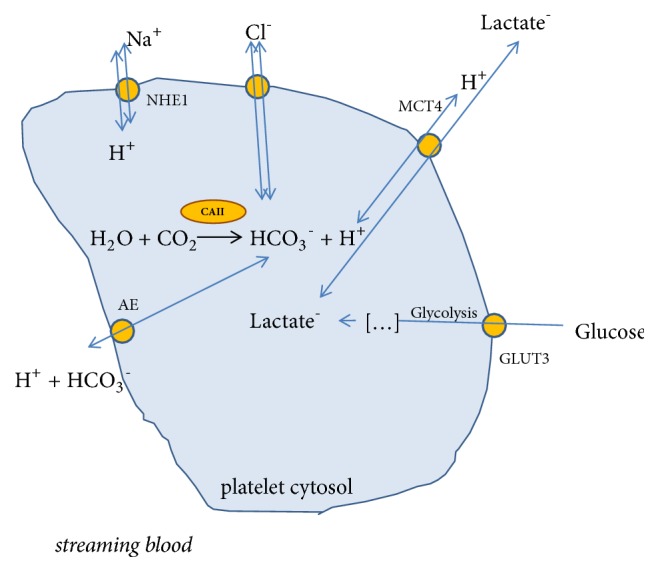
A potential role of carbonic anhydrase in the regulation of platelet metabolism [30–32].* GLUT3: glucose transporter; MCT4: H+/monocarboxylate transporter; AE: Anion exchanger; NHE1: the Na+/H+ exchanger.*

The first statements of CAII in platelets were made 60 years ago [53] and its more accurate characterization was created in the '80s of the last century [54]. CAII, by catalysing the formation of H^+^ and HCO_3_^−^, reduces the cytosol pH of the platelet [55]. The reaction products can be excreted outside the plasma membrane – H^+^ is being exchanged for Na^+^ whereas HCO_3_^−^ for Cl^−^ (Figure 2).

The exact role of CAII in platelet physiology requires more detailed research. A question arises whether their products play a direct role in platelet physiology or enhance the transmembrane ion exchange, sodium and chloride influx. So far there has been no clinical case presented in the PubMed database that describes CAII deficiency in platelets and, consequently, the effects of this deficiency on the phenotype. The only available case is the description of a murine mutation located in the direct proximity to the CAII gene locus, which affected both platelet morphology and function [56].

The CAII activity in platelets has been hypothesized since Akkerman et al. described proton efflux from platelets following thrombin stimulation [57, 58]. Siffert et al. verified the presence of CO_2_ hydration in platelets, subsequently proved that this process may be inhibited by ethoxzolamide, and showed that examined enzyme kinetics strictly corresponds with carbonic anhydrase II [54]. Afterwards, ethoxzolamide was shown to reduce the thrombin stimulated aggregation by about 20%-40% [59, 60]. Similar result was achieved by the removal of CO_2_ from platelets environment, which additionally confirms the importance of carbon dioxide hydration in the aggregation process [59].

### 3.1. Effect of Carbonic Anhydrase Activators on Platelet Function

Adrenaline is CAII agonist [61] and it can sensitize platelets to thrombin by induction of CAII activity [62, 63]. Adrenaline stimulates platelet carbonic anhydrase directly or by enhancing the membrane HCO_3_^−^/Cl^−^ exchange since such subthreshold doses of adrenaline (in concentrations which do not initiate aggregation) added to platelets are capable of increasing the chloride concentration in platelet cytosol. [55]. Not only does a CAII inhibitor, acetazolamide, reduce resting and adrenaline-induced chloride concentration in platelets, but it also abolishes the synergism of thrombin and low concentration of adrenaline [63]. The proaggregatory effect of adrenaline is also abolished by another CAII inhibitor, chlortalidone. Furthermore, the ability of adrenaline to initiate platelet aggregation has been demonstrated to be directly proportional to the activity of platelet carbonic anhydrase [64].

Serotonin is an activator of carbonic anhydrase [49], which is also a poor agonist for platelet aggregation [65]. Interestingly, it enhances adrenaline, adenosine diphosphate (ADP) and collagen induced aggregation [65]. Furthermore, while being hyperreactive to adrenaline alone or adrenaline+serotonin, platelets present increased binding affinity for serotonin [65]. This observation may be clinically important as in an* in vivo* dog model serotonin plasma level was increased in stress conditions and it was suspected to significantly interplay with the evolution of an occlusive coronary thrombus [66], which was verified in human observation trial where high plasma serotonin level was associated with presence of coronary artery disease and future cardiac events [67]. This effect seems to be mediated through platelet 5-HT (5-hydroxytryptamine) receptors as saprogelate (a 5-HT_2_ receptors antagonist) limits platelet aggregation [68–70]. To the best of our knowledge, the possible participation of direct serotonin influence on platelet carbonic anhydrase has not been evaluated.

The selective serotonin reuptake inhibitors (SSRI), fluoxetine, sertraline, and citalopram, are potent activators of carbonic anhydrase II [71] and therefore they may be suspected of proaggregatory properties. Nevertheless, another SSRI, paroxetine, decreases intraplatelet serotonin storage and at the same time lowers platelet activation [72]. Similarly, sertraline and citalopram also limit platelet aggregation [73, 74]. Fluoxetine was to decrease platelet aggregability in the case of a 49-year-old man [75]. Therefore, SSRI effect on platelet function may be the balance between their direct action on platelets and the result of decreased platelet serotonin content. Nevertheless, the impact of this group of drugs on both thrombotic [76] and bleeding events is not substantial [77].

Histamine is a poor carbonic anhydrase II activator [52] present in humans platelets and it plays an important role in modulating platelet function. Histamine does not initiate platelet aggregation by itself, but it increases platelet sensitivity to aggregation agonists like ADP, thrombin, collagen, arachidonic acid [78], and adrenaline [79]. In a clinical model of increased histamine release, patients with chronic urticaria, higher platelet activity was observed when compared to healthy controls: increased levels of soluble P-selectin in one study [80] and both enhanced aggregation in response to ADP and increased soluble P-selectin levels compared to healthy population in another paper (both parameters were strongly and positively correlated with the Urticaria Severity Score) [81]. The mechanism of histamine impact on platelet pathophysiology is not fully understood. Histamine proaggregatory properties seem to result from H_1_ receptor stimulation (located in platelet membrane [82]). However, involvement of histamine in intracellular metabolism is also suggested [78], which was the subject matter of some studies [79, 83, 84]. Interestingly, there are no studies verifying whether CA inhibitors attenuate histamine affect platelet aggregation and if such an inhibition may be useful, e.g., in chronic urticaria management.

Histidine, phenylalanine, and carnosine (*β*-alanine-histidine dipeptide) are carbonic anhydrase activators [52]. Data regarding effect of histidine and carnosine on platelet function are not consistent. In some studies, histidine was verified to be an inhibitor of ADP-induced platelet aggregation [85] or spontaneous platelet aggregation and thromboxane B_2_ (TxB_2_) formation [86], but in others it demonstrated both slight proaggregative activity (50% of the patients) and antiaggregatory activity (the other 50% of patients) [87]. Similarly, in one study carnosine slightly stimulated ADP-induced aggregation [87], but in another it enhanced platelet aggregation only in patients with a low rate of aggregation, but inhibited platelet aggregation in patients with high index of aggregation [88]. There are hardly any available data on phenylalanine influence on platelet function; in a single study phenylalanine methyl ester inhibited ADP-induced platelet aggregation. Interestingly, similar results were observed with histidine methyl ester, but not with pure histidine (no inhibition) [89].

There are no available papers analyzing histidine, phenylalanine, and carnosine influence on CAII in platelets. Nevertheless, while analyzing their action profile, they do not appear to act on platelets through CAII.

### 3.2. Diuretics as CAII Inhibitors

Systemic use of acetazolamide is dedicated for altitude sickness treatment and prevention. Primarily, it reverses hypocapnic alkalosis occurring due to ventilatory response to hypoxemia, but the exact mechanism of its beneficial action seems to be more complex and is not fully recognized [41]. Moreover, in people exposed to high altitudes, thrombosis is a proposed mechanism of several complications as in autopsy many megakaryocytes were present in the lungs of people diagnosed with pulmonary edema and in one case they were also accompanied by thrombi in the kidneys and liver [90]. Nevertheless, at high altitude platelets present increased adhesiveness [91], but decreased aggregation in response to ADP, adrenaline, and collagen [92, 93] so eventual benefit of acetazolamide antiaggregatory properties remains uncertain.

Diuretics commonly used in clinical practice (hydrochlorothiazide, chlortalidone, indapamide, furosemide, and bumetanide) are well-established carbonic anhydrase inhibitors [51]. Thiazide-like diuretics, indapamide and chlortalidone, are widely used in cardiovascular medicine. They are proved to prevent cardiovascular events (CVE) and reduce all-cause mortality. The CVE prophylactic effect exceeds the benefits of lowering blood pressure. The difference was revealed when this thiazide-like diuretics were compared with thiazide-type diuretics [94]. The important differences in activity between these two groups of drugs regard platelet inhibition, which is much more intense in thiazide-like than thiazide-type diuretics as shown in work by Rendu at al. comparing indapamide and hydrochlorothiazide properties [95]. Furthermore, the platelet inhibition by thiazide-like diuretics seems to be carbonic anhydrase dependent [64].

Loop diuretics, furosemide and bumetanide, are loop diuretics potent to inhibit carbonic anhydrase II activity [51]. In one clinical study, furosemide was shown to inhibit ADP-induced platelet aggregation both ex vivo and after intravenous infusion [96] and in another study furosemide inhibited* in vitro* both ADP- and AA-induced aggregation [97]. Bumetanide was also shown to inhibit adrenaline-induced aggregation [63]. The mechanism of influence of loop diuretics on platelets remains unclear and the involvement of carbonic anhydrase may be expected.

### 3.3. Physiological Nitrogen Compounds versus Platelet Carbonic Anhydrase II Function

Platelet aggregation may be inhibited by nitric oxide (NO). However there is a controversy about possible significance of this dependence since the extra-platelet NO concentration is low and it seems to be no expression of nitric oxide synthase in platelets [33, 34]. Nevertheless, the effect of NO on platelets is mediated through the activation of guanylyl cyclase to produce cyclic guanosine monophosphate (cGMP) [98, 99]. Interestingly, carbonic anhydrase II was found to catalyse generation of nitric oxide form nitrite [100], which may be an additional origination of intra-platelet NO. This reaction is significantly enhanced by both decreased pH and dorzolamide, which are inhibitors of CAII main activity [100]. On the other hand, other research groups provide evidence negating this CA activity [36].

Nitrite is a platelet inhibitor in whole blood [101], but added to platelets suspended in platelet rich plasma it does not inhibit platelet aggregation [102] or activate soluble guanylate cyclase (sCG) [36]. It does not produce increased intraplatelet nitrite concentration [103], either. Therefore it (in these conditions) cannot rich cytosolic enzymes like carbonic anhydrase II.

**Figure 3:**
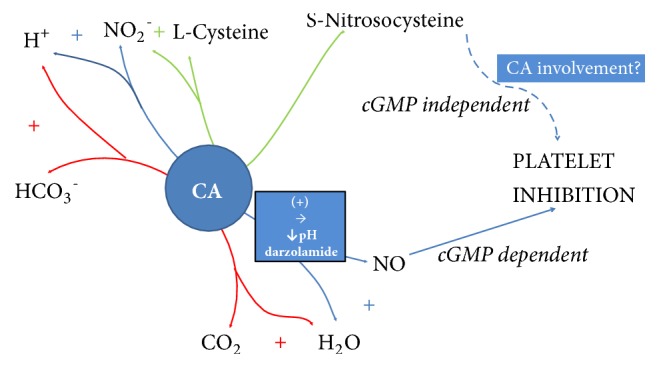
Postulated map of carbonic anhydrase II activities involving nitrosothiols, nitrate, and nitric oxide metabolism and they influence on platelet aggregation. S-Nitrosocysteine and NO are established platelet aggregation inhibitors [33, 34]. S-Nitrosocysteine is able to act independently of cGMP formation [35]. Dorzolamide and decreased pH increase nitric oxide formation [36]. CA ability to form NO from nitrite inside platelets remains controversial [36]. Nevertheless, NaHCO_3_^−^ + NO_2_^−^ in presence of erythrocytic CA II provides platelet cGMP formation [36] (more detailed description in the main text).

Platelet supplementation with S-Nitrosocysteine (SNC), a NO donor, also provides intraplatelet NO_2_^−^ formation and leads to platelet sCG activation [35, 36, 103]. Interestingly, SNC aggregation inhibition is not mediated by sCG activation. SNC also presents direct cyclooxygenase (COX) inhibiting properties, but an involvement of one more mechanism is postulated as it produces complete blockage of AA-induced aggregation, when TxB_2_ formation is only partially decreased [103]. Therefore, SNC is supposed to be involved in one more mechanism modulating platelet function, which may be CA. This thesis of CA contribution may be supported by observation that SNC produces increased intraplatelet nitrite formation [103]. CA II is proven to catalyse opposite reaction; it can produce S-nitrosothiols from inorganic nitrite [36] (Figure 3.).

Nitrate is able to bind to carbonic anhydrase active site [104], but not it cannot be utilized to form S-nitrosothiols as nitrite can be [105]. It can inhibit platelet aggregation in whole blood, but the mechanism remains unclear [101].

### 3.4. Other Drugs Potentially Inhibiting Platelet Carbonic Anhydrase II

An experiment considering influence of N-hydroxyurea on platelet activity was performed by Lahiri at al. in chronic myelogenous leukaemia. There were at least three patterns of response to hydroxyurea, no response, partial aggregation inhibition, and “de-aggregation”. Interestingly, both aggregation inhibition and “de-aggregation” were only partially reversible by ODQ (1H-(1,2,4)Oxadiazolo(4,3-a)quinoxalin-1-one), which indicates involvement of other mechanism than postulated by authors NO release stimulating cGMP formation [106]. The inhibition of platelet carbonic anhydrase II by N-hydroxyurea may be possible [107].

Coumarin and its derivates are carbonic anhydrase inhibitors and substrates [108, 109]. Unfortunately, there is no information on acenocoumarol and warfarin (two coumarin derivates widely used as anticoagulant drugs [110]) regarding their individual influence on carbonic anhydrase. Nevertheless, these two drugs do not seem to inhibit platelet carbonic anhydrase in humans as both of them enhance platelet activity [111, 112].

**Figure 4:**
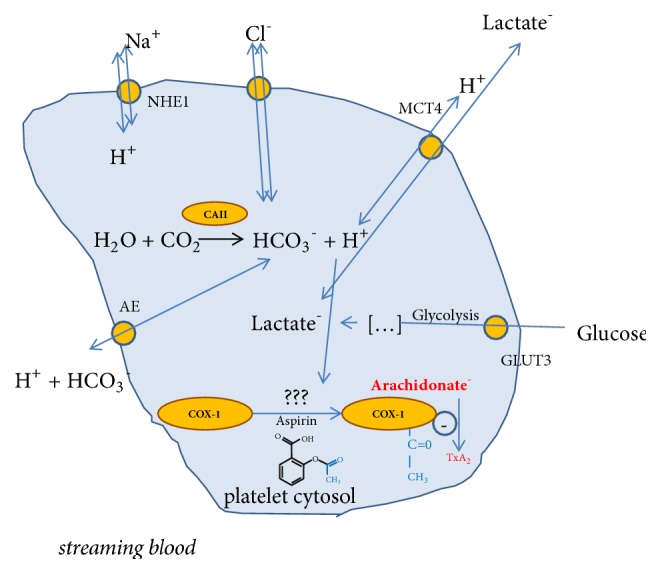
A potential role of carbonic anhydrase II (CAII) in regulating ASA response [30–32, 37].* GLUT3: glucose transporter; MCT4: H+/monocarboxylate transporter; AE: anion exchanger; NHE1: the Na+/H+ exchanger; COX-1: cyclooxygenase-1; TxA*_*2*_*: thromboxane A*_*2*_.

A substantial part of nonsteroid anti-inflammatory drugs (NSAIDs) present anti-CAII activity. Celecoxib [113, 114] and valdecoxib [115], but not rofecoxib [114], meloxicam, piroxicam, and lornoxicam [116], are carbonic anhydrase II inhibitors. Diclofenac, which does not possess sulphonamide moiety, is not active against CA [116]. Flurbiprofen is a weak carbonic anhydrase II inhibitor whereas ibuprofen and indomethacin are even much less active against this enzyme [117]. In two papers by Puscas et al. indomethacin was said to be an agonist of CA, but this conclusion was reached after indomethacin limited inhibitory effect of acetazolamide on carbonic anhydrase [118, 119] and it was uncertain whether it was indeed CA agonist or just weaker than acetazolamide CA inhibitor competing for a binding site. Acetylsalicylic acid (ASA) is a noncompetitive carbonic anhydrase II inhibitor [120], but this activity may be associated with adjusting environmental pH by ASA [121]. The effect of other NSAID on platelet function does not seem to depend on CAII regulation, but exclusively on COX-1 inhibition. A nonspecific COX inhibitor naproxen, but not COX-2 selective inhibitor celecoxib inhibits both collagen and arachidonate induced platelet aggregation [122]. Nevertheless, in a recent report we provided evidence that interindividual variability in* in vitro* platelet responsiveness to acetylsalicylic acid may be associated with carbonic anhydrase II concentration. Briefly, ASA low-responders presented increased intraplatelet CAII concentration and more intense baseline arachidonic acid induces aggregation compared to ASA sensitive individuals [123]. Among the CAII-dependent mechanisms modifying the platelet responsiveness, the pH changes of platelet cytosol leading to impaired acetylating of cyclooxygenase by ASA are noteworthy. This in turn could affect the antiplatelet effect of ASA as well as platelet inflammatory activity and energetic metabolism (Figure 4).

To conclude, the role of carbonic anhydrase II in cardiovascular medicine is still underestimated and requires further in-depth studies.

## 4. Conclusions

Even though there is a well-documented rationale for an important role of carbonic anhydrase II in regulating platelet function, its exact role in platelet physiology and pathology remains poorly understood. A more frequent use of platelet CAII inhibitors holds promise as a good strategy to reduce the impact of cardiovascular diseases. However, future prospective clinical studies, supported by the evidence-based medicine principles, are needed in order to precisely elucidate the role of platelet CAII in cardiovascular medicine.

There is a need for more basic scientific investigations in order to establish the role of platelet carbonic anhydrase II in the pathogenesis of several diseases such as chronic urticaria and altitude sickness and, further, to verify the contribution of platelet CAII in metabolism of nitrites and antiaggregatory properties of S-Nitrosocysteine.
